# Continuous Ethanol Fermentation of Pretreated Lignocellulosic Biomasses, Waste Biomasses, Molasses and Syrup Using the Anaerobic, Thermophilic Bacterium *Thermoanaerobacter italicus* Pentocrobe 411

**DOI:** 10.1371/journal.pone.0136060

**Published:** 2015-08-21

**Authors:** Rasmus Lund Andersen, Karen Møller Jensen, Marie Just Mikkelsen

**Affiliations:** Estibio ApS, Ballerup, Denmark; University of Huddersfield, UNITED KINGDOM

## Abstract

Lignocellosic ethanol production is now at a stage where commercial or semi-commercial plants are coming online and, provided cost effective production can be achieved, lignocellulosic ethanol will become an important part of the world bio economy. However, challenges are still to be overcome throughout the process and particularly for the fermentation of the complex sugar mixtures resulting from the hydrolysis of hemicellulose. Here we describe the continuous fermentation of glucose, xylose and arabinose from non-detoxified pretreated wheat straw, birch, corn cob, sugar cane bagasse, cardboard, mixed bio waste, oil palm empty fruit bunch and frond, sugar cane syrup and sugar cane molasses using the anaerobic, thermophilic bacterium *Thermoanaerobacter* Pentocrobe 411. All fermentations resulted in close to maximum theoretical ethanol yields of 0.47–0.49 g/g (based on glucose, xylose, and arabinose), volumetric ethanol productivities of 1.2–2.7 g/L/h and a total sugar conversion of 90–99% including glucose, xylose and arabinose. The results solidify the potential of *Thermoanaerobacter* strains as candidates for lignocellulose bioconversion.

## Introduction

As yeasts have been used for ethanol production for thousands of years they are also considered to be the obvious candidate for lignocellulosic ethanol production. The high ethanol tolerance of yeast and high yields of ethanol obtained in starch or sucrose derived fermentations are unsurpassed by any other microorganism and the combination of a low pH during fermentation and high ethanol titers assist in preventing contamination. Lignocellulosic hydrolysates, however, create a completely different fermentation environment as they contain organic inhibitors, including acetic acid and phenolic lignin degradation products, and significant amounts of sugars that are not naturally fermented by industrial ethanol-producing yeasts. Today, 20 years after the first patent application claiming a xylose-utilizing *Saccharomyces cerevisiae* was filed [1], development of yeast strains able to ferment all hexose and pentose sugars in non-detoxified lignocellulosic hydrolysates still remains a major challenge [2]. For example some sugars are metabolized sequentially due to metabolic regulation and competition for transporters; xylose is still metabolized at a slower rate than glucose and high xylose/glucose ratios can affect the tolerance of yeast to inhibitors. To our knowledge, the combination of an ethanol titre of above 45 g/L and an overall yield of more than 90% of theoretical (0.46 g ethanol per g available sugar, not including arabinose) has not been described using a genetically modified yeast growing on non-detoxified lignocellulosic hydrolysates with no additional sugar additions [2].

It has been demonstrated that many thermophilic bacteria are highly efficient ethanol producers, with the natural ability to metabolize both pentoses and hexoses found in lignocellulosic hydrolysates. The advantages of using these thermophilic bacteria include the prevention of contamination from mesophilic bacteria and fungi due to high temperature fermentation, energy savings as cooling after enzymatic hydrolysis and during fermentation is avoided and a broad sugar metabolism spectrum that enables the use of almost all sugars in the biomasses [3–7].

It has been shown that the *Thermoanaerobacter* BG1 can grow and produce ethanol from hemicellulose hydrolysate of wheat straw and corn stover with the same ethanol yield as with synthetic medium [4,5]. To increase the ethanol yield of this organism, lactic acid as well as acetic acid production has been eliminated by knocking out the genes encoding Lactate dehydrogenase, Phosphotransacetylase and Acetate kinase, resulting in the strain *Thermoanaerobacter italicus*—Pentocrobe 411 (PC 411) [8].

In this paper, data from continuous fermentation studies on a range of different biomasses using Pentocrobe 411 are presented showing the organism’s ability to grow on non-detoxified complex biomasses with high dry matter concentrations and produce ethanol with high titer, yield and productivity.

## Materials and Methods

### Organism

Pentocrobe 411, is a genetically modified version of the strictly anaerobic thermophilic bacterium *Thermoanaerobacter italicus* BG10 (German Collection of Microorganisms and Cell Cultures, DSM 23015) [9], with a deletion of the genes encoding Lactate dehydrogenase (LDH), Phosphotransacetylase (PTA) and Acetate kinase (AK) [8]. Pentocrobe 411 is available from the German Collection of Microorganisms and Cell Cultures (DSM) with identification number DSM 29083. The deposited strain, Pentocrobe 411X, is adapted to growth on xylose containing medium. A genetically identical strain adapted to growth on sucrose by 10 serial transfers to sucrose containing minimal medium, Pentocrobe 411S, was used for fermentations on sugar cane syrup and molasses. An antibiotic resistance marker free version of Pentocrobe 411, Pentocrobe 463, can be purchased from Estibio ApS, Denmark.

### Culture medium and nutrients

Batch cultures and batch reactor start-up was performed in BA medium [10] with the following modifications: cysteine and the vitamin solution were omitted, the concentration of sodium selenite was decreased to 0.06 g/L, and the Iron (II) chloride was increased to 2.3 g/L.

For continuous fermentations, increasing amounts of NH_4_^+^, Mg^2+^, Ca^2+^, PO_4_^3-^ were added in correspondence with the sugar concentrations to maintain the salt concentration in the reactor similar to the batch medium described above. The concentrations of salts were regularly monitored as described below. During continuous fermentation of pretreated wheat straw, the medium was supplemented with 0.037 gram of yeast (25% yeast extract (N1 OrganoTechnie 10284) and 75% dried full yeast (Springaline, Bio Springer)) and 0.024 gram of centrifugally separated corn steep liquor (CSL, Solulys 048E) per gram of sugar and 3 g/L raw glycerol (Emmelev biodiesel plant, Denmark).

For reactor batch start-up, 15g/L xylose (Danisco, Denmark) was added to the modified BA medium described above. During the continuous fermentations the liquid fraction of the pretreated biomasses was supplemented with minerals, trace metals, yeast and corn steep liquor as specified above. The composition of the pretreated biomasses used for the fermentations is shown in Table 1. The feed materials were autoclaved together with the BA components (@121°C, 20 min). Carbon- and nitrogen sources were autoclaved separately to prevent Maillard reactions.

**Table 1:** Composition of undiluted, pretreated biomasses used for fermentation influents.

Experiment name	Biomass	Pretreatment	Sugar_Total_ (g/L)	Glucose (g/L)	Xylose (g/L)	Arabinose (g/L)	Sucrose (g/L)	Acetic acid (g/L)	Lactic acid (g/L)	Furfural (g/L)
WS80	Wheat straw	Dilute acid	65	9	51	5	0	6	0	0.5
WS97-N2	Wheat straw	Dilute acid	137	89	48	6	0	7	0	0.4
WS10	Wheat straw	Dilute acid	77	15	60	2	0	3	0	1.0
BI13B	Birch	Dilute acid	86	50	36	0	0	6	0	0.2
CC02-N2	Corn cob	Dilute acid	134	76	52	6	0	6	0.5	0.4
SB10B	Cane bagasse	Sulfite	436	276	148	12	0	0.8	20	1.4
CA20-1	Cardboard	Enzymatic	56	42	14	0	0	0.5	14	0.1
BW20-2	Biowaste	Enzymatic	75	39	36	0	0	2	7	0
EFB16-1-N2	Oil palm EFB	Dilute acid	190	106	80	4	0	9	0.6	1.2
FR16-2-N2	Oil palm frond	Dilute acid	130	73	52	5	0	5	0	1.0
CS02B-N2	Cane Syrup	None	868	n.a.	n.a.	20	848	1	9	0
CM02B-N2	Cane Molasses	None	878	n.a.	n.a.	15	864	3	13	0

Concentrations of carbohydrates, acids, and furfural in the pretreated biomasses before neutralization and dilution (g/L). All biomasses were diluted, pH adjusted, and added nutrients before fermentation. The total sugar in the resulting influents at the time of data collection can be found in Table 2.

0.1% and 0.2% Antifoam 204 (Sigma-Aldrich) was used at sugar concentrations below and above 100 g/L respectively.

### Culture conditions

The experiments were performed in temperature controlled stirred laboratory fermentors with working volumes of either 225 (no nitrogen sparging) or 450 mL (with nitrogen sparging). The systems were equipped with pH and temperature sensors and nitrogen sparging was implemented to reduce the reactor ethanol concentration when influents with a sugar concentration of above 60 g/L were employed. The fermentations were performed at 66°C, pH 7. Pictures of the fermentation system are shown in S1 Fig.

Seed cultures were prepared by inoculating 20 ml Hungate tubes containing 10 ml anaerobic modified BA medium, as described above, with 5 g/L xylose with either 100 μL Pentocrobe from a -80°C freezer culture or 100 μL from a continuously operating fermentor. The tubes were incubated overnight at 65°C.

Sterile batch medium containing xylose was transferred aseptically to the fermentors. Before seed culture transfer the temperature was increased to 66°C, the medium was gassed for 5 minutes with N_2_/CO_2_ (80/20%), and Na_2_S (0.05g/L) was added to ensure anaerobic conditions, and the fermentors were inoculated with 1–5% v/v seed culture. Approximately 8–18 hours after inoculation, after a decrease in pH to below 6.5 was observed and culture turbidity was visibly increased (OD600 > 0.1), the initial feed was started.

During continuous fermentation the influents were not added Na_2_S and were not kept anaerobic. The gas production in the reactor reduces the oxygen tension in the reactor to a tolerable level. Throughout the fermentation, the feed rate was continuously changed in response to optical density and sugar concentrations in the fermentation broth to keep the optical density and sugar conversion stable. When increasing influent sugar concentrations, the feed rate was decreased to maintain constant ethanol productivity.

### Analytical methods

#### High Performance Liquid Chromatography (HPLC)

Cellobiose, glucose, xylose, arabinose, glycerol, lactate, acetate, ethanol, HMF and 2-furaldehyde were quantified by HPLC-RI using a Dionex Ultimate 3000 (Dionex corp.) fitted with a Rezex ROA column (300 X 7.8mm) (Phenomenex, USA) combined with a SecurityGuard Cartridge Carbo-H 4*3.0mm (Phenomenex, USA). The analysts were separated isocratically with 4.5 mM H_2_SO_4_ at 60°C for 30 or 45 minutes.

#### Optical density and cell dry mass

Cell growth was determined by measuring optical density (OD) at 600 nm. When calculating cell dry mass, a conversion factor of 0.36 [(g/L) dry cell weight/OD600] was used (based on own results).

#### NH_4_ analysis

The NH_4_ concentration in the fermentation broth was monitored using the spectrophotometrical assay kit Ammonium cuvette test 0.015–2.0 mg/L NH4-N (Hach Lange).

#### Estimation of ethanol loss

The redox and carbon balance were used to determine ethanol loss and to correct for ethanol loss due to evaporation [5]. The ethanol loss determinations were validated by experimental determination of ethanol evaporation as described previously [5] and by ethanol condensation of the gas phase.

#### Statistical design of experiments for analysis of inhibitory effect

Duplicates of 20 different variations of media containing from 0–5 g/L acetic acid, 0–1 g/L furfural and 5, 10, or 15 g/L xylose were fermented using Pentocrobe 411 in closed 10 mL anaerobic tubes (40 fermentations total). The pressure of each tube was measured after 48 hours as an indication of growth. The data were analyzed using the design of experiments software package MODDE (Umetrics).

## Results

### General

*Thermoanaerobacter italicus* Pentocrobe 411 was tested in continuous fermentations of different lignocellulosic substrates to investigate the limits to ethanol productivity, ethanol concentration and yield. All experiments were performed in continuous culture as the productivity and tolerance of *Thermoanaerobacter* sp. have been demonstrated to be significantly higher in continuous as compared to batch culture. The biomasses were pretreated by BioGasol, Ballerup, Denmark using dilute acid catalyzed steam explosion and were then either 1) enzymatically hydrolyzed to release glucose or 2) the xylose, arabinose, and inhibitor-containing liquid fraction of the biomass was supplemented with glucose to simulate enzymatic hydrolysate. The compositions of the resulting undiluted, pretreated biomasses are shown in Table 1.

### Continuous fermentation of wheat straw hydrolysate using Pentocrobe 411

**Fig 1:**
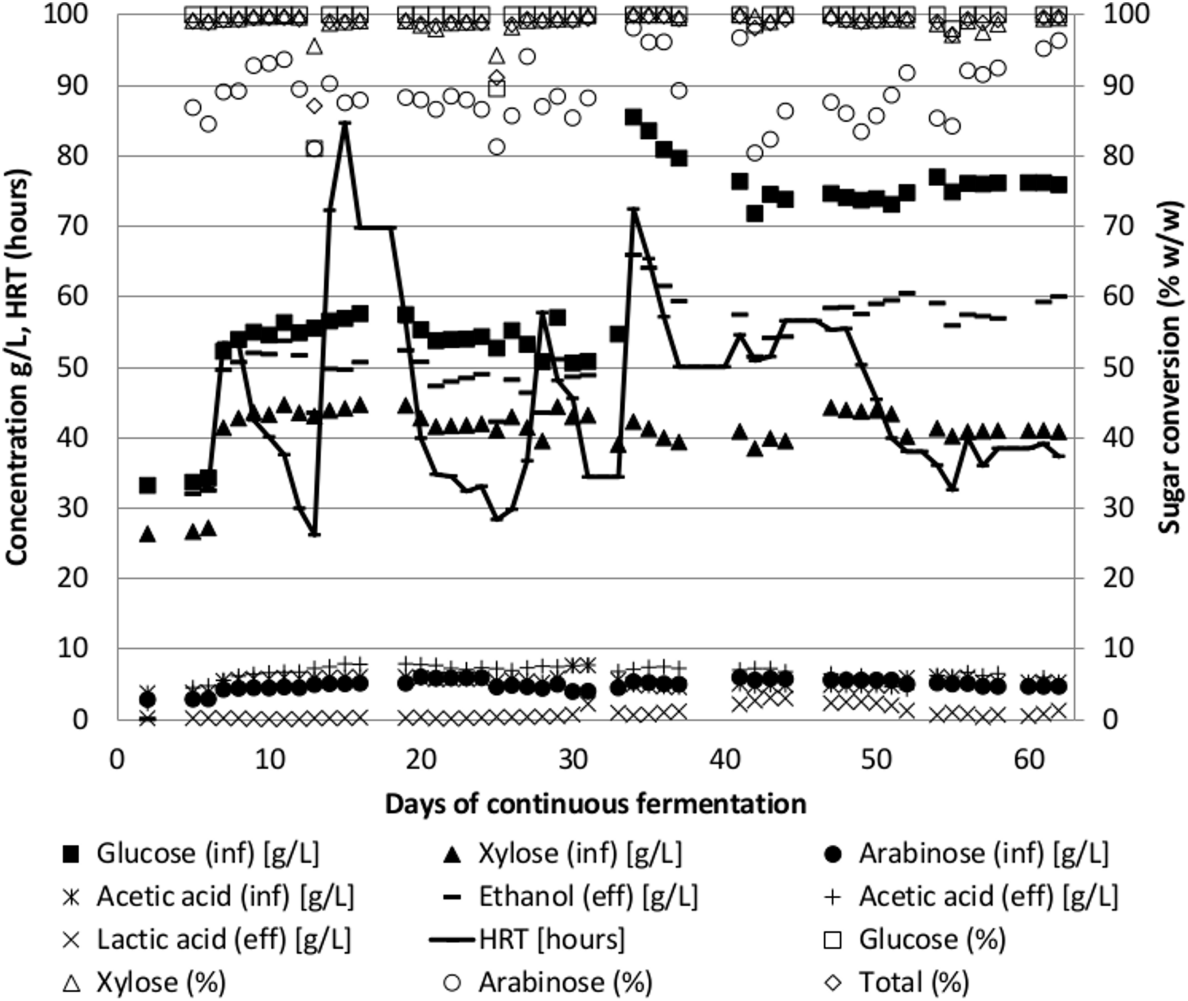
Time course of continuous fermentation of un-detoxified wheat straw hydrolysate using the thermophilic anaerobic bacterium *Thermoanaerobacter* Pentocrobe 411. Inf: HPLC data from the influent bottle. Eff: HPLC data from the reactor effluent. Ethanol (eff) is a combination of ethanol in the reactor effluent and ethanol from the gas phase exiting the reactor system. The conversion of sugars in % is based on the concentration in the reactor relative to the concentration in the influent. HRT: Hydraulic retention time.

A continuous reactor was set up to test the Pentocrobe 411 limits to ethanol concentrations and productivity on pretreated and enzymatically hydrolyzed wheat straw (Fig 1 and Table 2, WS97-N2). The reactor startup was performed in batch. After 15 hours of batch fermentation, the first influents were started at a flow rate of 333 mL/day. Nitrogen gas stripping was started at a flow rate of 0.2 L/min 24 hours after initiation of the influent. The first fermentation influent contained 62 g/L total sugar, comprising 33 g/L glucose, 26 g/L xylose and 3 g/L arabinose, corresponding to 10% (w/w) wheat straw dry matter after pretreatment.

**Table 2:** Overview of results from continuous fermentations using *Thermoanaerobacter* Pentocrobe 411X and 411S.

Experiment name	Reactor type	Material	Volume (L)	C_sugar(max)_ (g/L)	C_EtOH_ (g/L)	Q_EtOH_ (g/L/h)	Y_EtOH(GXA)_ (g/g)	Y_EtOH(GX)_ (g/g)	DM_max_ (%)	Conv_(GXA)_ (%)	HRT (h)	Organism
WS80	CSTR	Wheat straw (pentose liquid)	0.5	53	25	2.1	0.47	0.48	15	90	12	PC 411X
WS97-N2	CSTR-N2	Wheat straw	0.5	121	58	1.5	0.48	0.50	19	99	38	PC 411X
WS10	CSTR	Wheat straw (pentose liquid)	75	68	32	1.3	0.47	0.48	15	97	25	PC 411X
BI13B	CSTR	Birch	0.5	60	28	1.5	0.47	0.48	10	99	19	PC 411X
CC02-N2	CSTR-N2	Corn cob	0.5	98	45	1.6	0.47	0.50	15	98	28	PC 411X
SB10B	CSTR-N2	Cane bagasse	0.5	214	103	1.8	0.48	0.49	n.t.	99	59	PC 411X
CA20-1	CSTR	Cardboard	0.5	49	23	1.4	0.47	0.47	n.t.	98	17	PC 411X
BW20-2	CSTR	Biowaste	0.5	73	35	1.2	0.48	0.48	n.t.	99	30	PC 411X
EFB16-1-N2	CSTR-N2	Empty fruit bunch	0.5	106	50	2.1	0.47	0.48	15	99	19	PC 411X
FR16-2-N2	CSTR-N2	Oil palm frond	0.5	94	46	1.6	0.49	0.51	18	98	29	PC 411X
CS02B-N2	CSTR-N2	Cane Syrup	0.5	170	80	2.0	0.47	0.47	n.a.	97	40	PC 411S
CM02B-N2	CSTR-N2	Cane Molasses	0.5	217	104	2.7	0.48	0.48	n.a.	94	39	PC 411S

**Fig 2:**
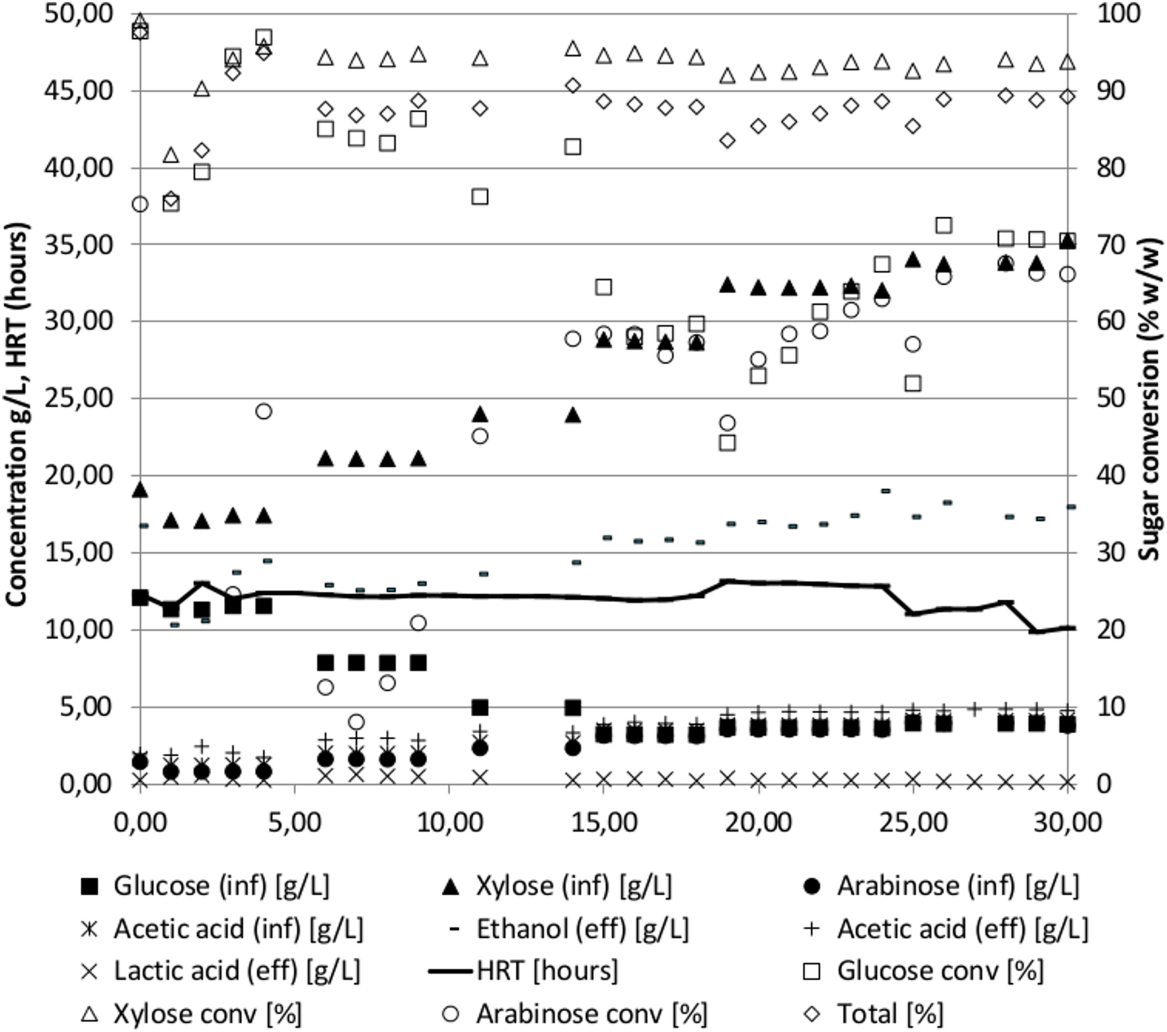
Time course of continuous fermentation of the un-detoxified pentose rich liquid fraction after separation of pretreated wheat straw using the thermophilic anaerobic bacterium *Thermoanaerobacter* Pentocrobe 411X. Inf: HPLC data from the influent bottle. Eff: HPLC data from the reactor effluent. Ethanol (eff) is a combination of ethanol in the reactor effluent and ethanol from the gas phase exiting the reactor system. The conversion of sugars in % is based on the concentration in the reactor relative to the concentration in the influent. HRT: Hydraulic retention time.

**Fig 3:**
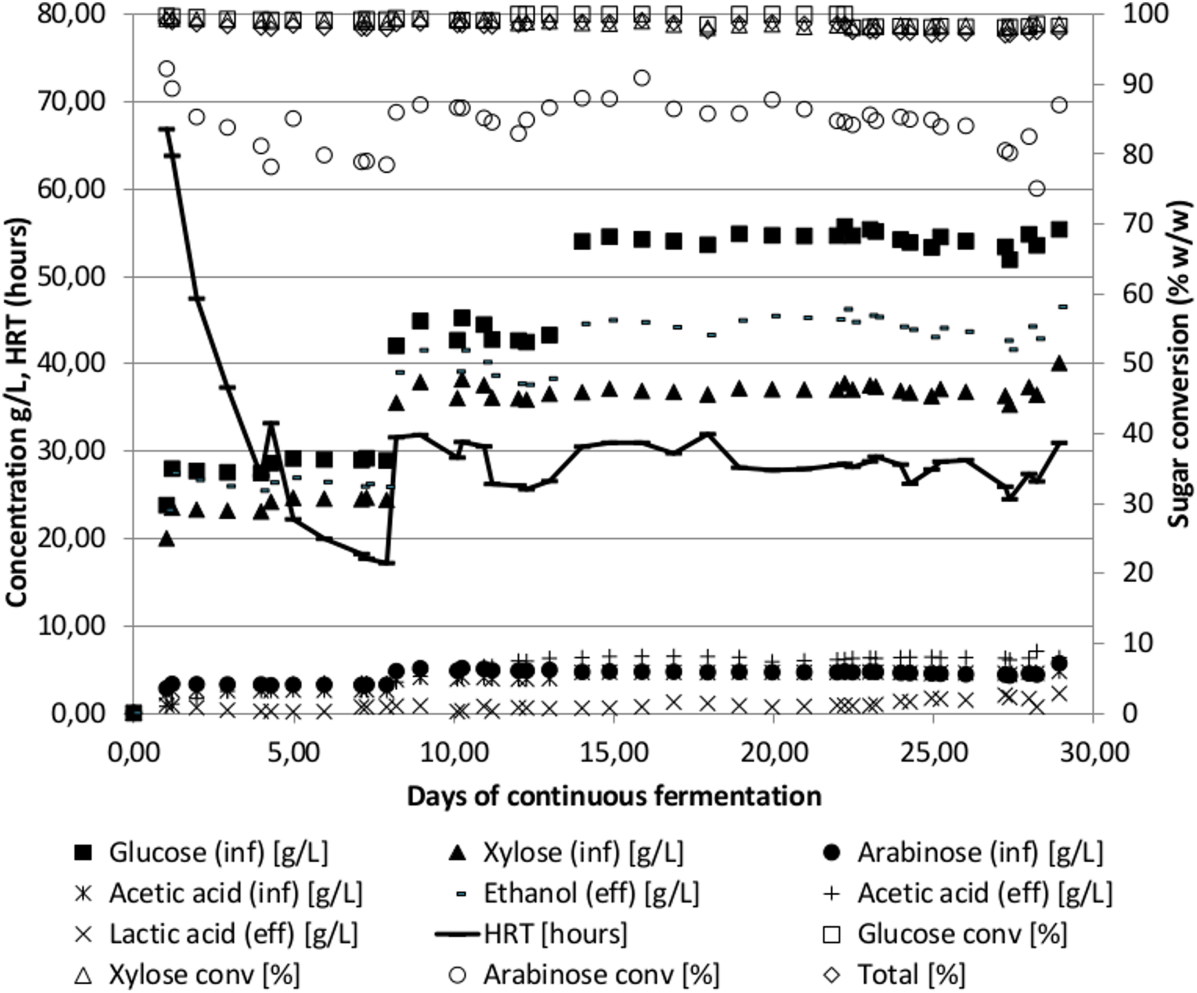
Time course of continuous fermentation of un-detoxified corn cob hydrolysate using the thermophilic anaerobic bacterium *Thermoanaerobacter* Pentocrobe 411X. Inf: HPLC data from the influent bottle. Eff: HPLC data from the reactor effluent. Ethanol (eff) is a combination of ethanol in the reactor effluent and ethanol from the gas phase exiting the reactor system. The conversion of sugars in % is based on the concentration in the reactor relative to the concentration in the influent. HRT: Hydraulic retention time.

**Fig 4:**
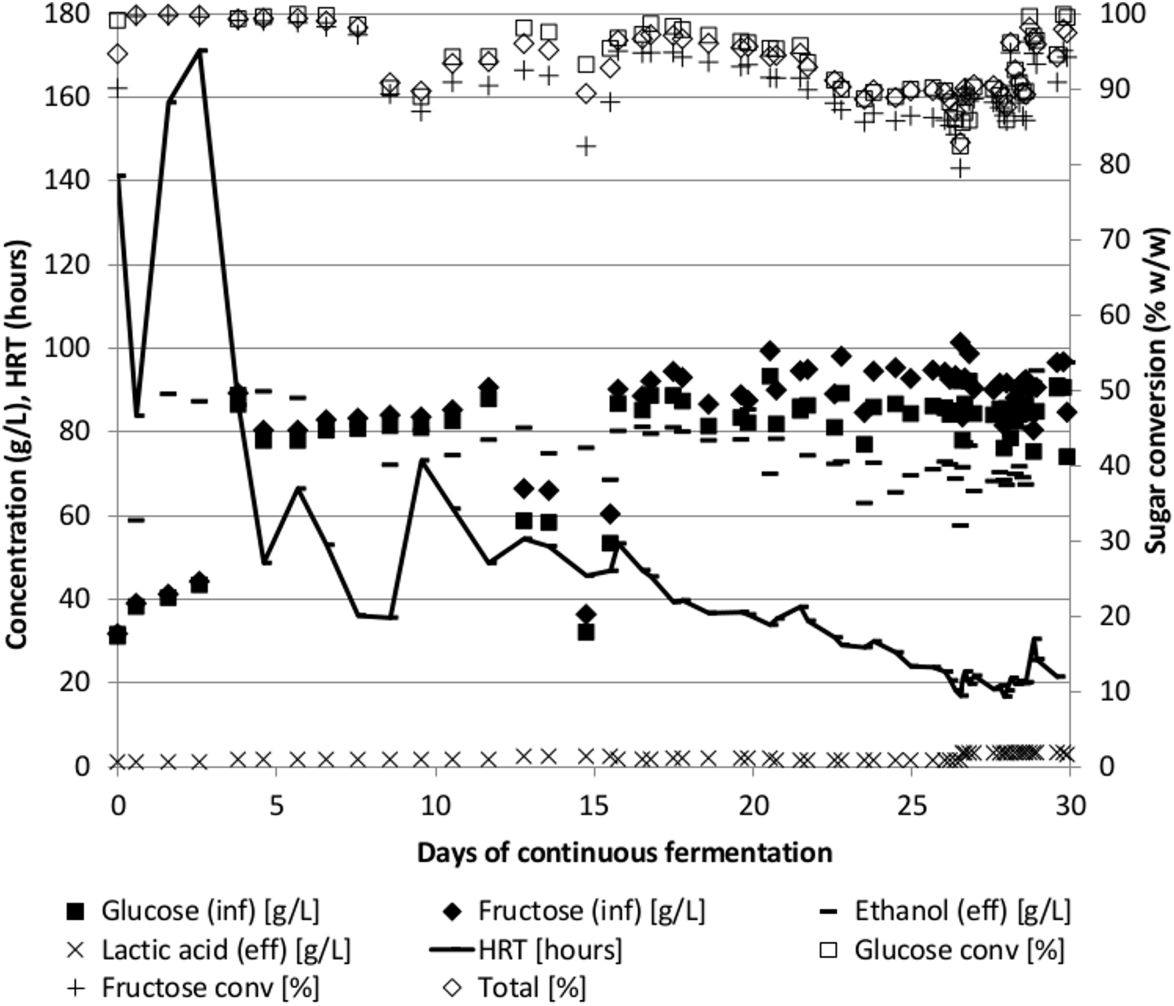
Time course of continuous fermentation of sugar cane syrup using the thermophilic anaerobic bacterium *Thermoanaerobacter* Pentocrobe 411S. Inf: HPLC data from the influent bottle. Eff: HPLC data from the reactor effluent. Ethanol (eff) is a combination of ethanol in the reactor effluent and ethanol from the gas phase exiting the reactor system. The conversion of sugars in % is based on the concentration in the reactor relative to the concentration in the influent. HRT: Hydraulic retention time.

The values are an average of data collected over five residence times at steady-state. C_sugar(max)_: Average total concentration of glucose, xylose, arabinose, and sucrose in the period of data collection. The ratio between sugars can be derived from Table 1. The time course profiles of the WS97-N2, WS80, CC02-N2, and CS02-N2 fermentations are shown in Figs 1, 2, 3, and 4, respectively. C_EtOH_: Concentration of ethanol corrected for loss of ethanol based on redox balance. Q_EtOH_: Volumetric ethanol productivity. Y_EtOH(GXA)_: Yield of ethanol based on influent glucose, xylose, and arabinose. Y_EtOH(GX)_: Yield of ethanol based on influent glucose and xylose. DM_max_: The biomass dry matter after pretreatment corresponding to the total sugar applied in the data collection period. Conv_(GXA)_: Conversion of glucose, xylose and arabinose in based on effluent levels relative to influent levels. HRT: Hydraulic retention time.

The results from the fermentation can be seen in Fig 1. The concentration of the pretreated wheat straw component of the influent was gradually increased to around 121 g/L total sugar where optimal performance was achieved. The feed rate was continuously adjusted to keep the total sugar conversion between 98% and 99% (converted/influent glucose, xylose and arabinose) and the resulting hydraulic retention time therefore varies throughout the fermentation. When a feed with a higher sugar concentration was initiated, the feed rate was reduced to allow adjustment to the new conditions (Fig 1, day 7 and 34). Also, when a decrease in sugar conversion was observed, the feed rate was decreased (Fig 2, day 13 and 25). Nitrogen sparging was adjusted to maintain reactor ethanol concentrations below 25 g/L. The gas phase from the reactor was condensed and sent to the reactor effluent to capture the ethanol from the gas stream. The ethanol concentration shown in Fig 1 is the combined ethanol from the effluent and the gas stream.

The acetic acid and lactic acid production was below 2 g/L throughout most of the fermentation showing the beneficial effect of the deletion of lactate dehydrogenase, phosphotransacetylase and acetate kinase in Pentocrobe 411. The increase in lactic acid seen on day 31 and between day 40 and 51 was due to a microbial contamination in the influent as similar amounts of lactic acid could be measured in the influent bottle. When a new influent was added on day 51, the effluent lactic acid concentration gradually decreased showing that the high temperature fermentation was able to continue even after difficulties were encountered with influent contamination. During the period of lactic acid contamination, the arabinose conversion decreased, however as soon as the contaminated influent bottle was changed the conversion increased indicating that the contaminating microorganism was imposing stress conditions on Pentocrobe 411.

On day 35, an influent with additional glucose was added to the reactor to test the performance at higher sugar concentrations. The ratio of glucose to xylose in this last influent was similar to the ratio in wheat straw, but as the enzymatic cellulose to glucose conversion was not complete, additional glucose was added to mimic full enzymatic conversion of the biomass. As can be seen from Fig 1, the organism responded well to the increased sugar concentration and the retention time could be reduced to around 38 hours by the end of the experiment, corresponding to a volumetric ethanol productivity of 1.5 g/L/h. In this last period of the fermentation, the ethanol concentration reached 58 g/L on average with a yield of 0.48 g/g (g of ethanol per g of influent glucose, xylose and arabinose). The sugar conversion in the final period was 99% and included glucose, xylose and arabinose.

### Fermentation of pentose rich liquid fraction of dilute acid pretreated wheat straw

In addition to the experiment described above, a fermentation of the pentose-rich liquid fraction of pretreated wheat straw was performed (WS80 in Tables 1 and 2, Fig 2). The biomass was pretreated similarly to the wheat straw experiment above (WS97-N2) but instead of performing enzymatic hydrolysis, the pentose rich liquid fraction was separated from the cellulose-rich solid fraction, the liquid fraction was adjusted to a neutral pH and used directly in the influents. The reactor was started and controlled similarly to the wheat straw hydrolysate experiment above (WS97-N2), except that nitrogen sparging was not employed. This influent was an example of a low value substrate containing mixed xylose and arabinose after extraction of the higher value cellulose fraction. Because the total sugar concentration was low due to the low level of glucose, the ratio of the inhibitors acetic acid and furfural to total sugar was almost twice that of the WS97-N2 hydrolysate. In spite of this high ratio of inhibitors to sugars, the ethanol productivity was among the highest observed for lignocellulosic biomasses (2.1 g/L/h). The ethanol yield of 0.47 g/g (glucose, xylose and arabinose) was similar to the yields obtained with hydrolysates with higher glucose/xylose ratios. The final ethanol concentration was lower than for WS97-N2 due to the lower influent sugar level and sugar conversion was somewhat lower (90%) most likely an effect of the high feed rate leading to high productivity. As can be seen from Fig 2, the xylose conversion is higher than the glucose conversion throughout the fermentation, suggesting that the concentration of the sugar is more important than the type (glucose, xylose, or arabinose). In the last 15 days of the experiment, the glucose and arabinose were similar and their conversion rates were almost identical. The fermentation of wheat straw pentose fraction (WS80) was repeated in a 75L pilot scale reactor with similar results, although the ethanol concentration was slightly higher, the conversion was higher and the productivity lower (Tables 1 and 2, WS10). The differences between the lab and pilot scale fermentations are most likely caused by a more conservative approach in the 75L pilot fermentation, where a higher hydraulic retention time was chosen to minimize the risk of wash-out, causing the lower productivity and higher sugar conversion in WS10 (pilot) as compared to WS80 (lab).

### Fermentation of other lignocellulosic biomasses

Similarly to the fermentation of wheat straw hydrolysate described above, five other lignocellulosic hydrolysates were tested namely; birch wood, corn cob, sugar cane bagasse, oil palm empty fruit bunch and oil palm frond (Tables 1 and 2). Except for sugar cane bagasse, all biomasses were pretreated using dilute acid steam explosion (hydrolysate supplied by BioGasol ApS, Ballerup, Denmark). The sugar cane bagasse was pretreated using a sulphite process and the lignin-containing liquid fraction was removed prior to enzymatic hydrolysis (hydrolysate supplied by BioGasol ApS, Ballerup, Denmark). The total sugar concentration in the influents varied from 86 to 436 g/L with glucose/xylose/arabinose ratios of 58/39/3 on average. The highest relative level of acetic acid relative to total sugar in the influent was observed for birch wood (7g/100g) while the highest lactic acid and 2-furaldehyde (furfural) concentrations were seen in cane bagasse and oil palm frond respectively (5g/100 g and 0.8g/100g respectively). The lactic acid in the cane bagasse is most likely due to contamination rather than being a product of pretreatment.

The fermentations of corn cob, oil palm empty fruit bunch, and oil palm frond resulted in similar performance with a maximum ethanol concentration of 94–106 g/L, a volumetric ethanol productivity of 1.6 to 2.1 g/L/h, an ethanol yield of 0.47–0.49 and a sugar conversion of 98–99% (glucose, xylose and arabinose). The time course profile of the fermentation of corn cob is shown in Fig 3.

The maximum ethanol concentration in the birch wood fermentation was 28 g/L, but as nitrogen sparging was not employed in this fermentation it may have been inhibited by the reactor ethanol concentration. Alternatively, the higher acetic acid concentration relative to total sugar or other biomass inhibitors may be inhibiting for the fermentation. The sulphite pretreated cane bagasse fermentation resulted in a higher ethanol concentration as compared to the dilute acid pretreated biomasses most likely due to a lower concentration of furfural and particularly acetic acid in the pretreated biomass. The maximal ethanol concentration reached was more than 100 g/L and a high productivity could be maintained at 1.8 g/L/h with a total sugar conversion of 99%.

### Fermentation of syrup and molasses

Sugar cane syrup and molasses were fermented similarly to the lignocellulose fermentations above using Pentocrobe 411 (Tables 1 and 2, CS02B-N2 and CM02B-N2, Fig 4). Prior to the fermentations, the strain was adapted to growth on sucrose by serial transfers to BA medium containing sucrose in batch culture and the adapted strain was named Pentocrobe 411S. As expected, higher ethanol concentrations and ethanol productivities could be obtained on syrup and molasses as compared to lignocellulosic hydrolysates. Molasses performed slightly better with an ethanol concentration of 104 g/L compared to 80 g/L for syrup and an ethanol productivity of 2.7 g/L/h compared to 2.0 g/L/h for syrup. The ethanol yields in the syrup and molasses fermentations were 0.47 and 0.48 g/g (glucose, xylose and arabinose) respectively which is comparable to the lignocellulose fermentations. The time course profile of the cane syrup fermentation is shown in Fig 4.

### Fermentation of other waste biomasses

Cardboard waste material and mixed food production waste hydrolysates are a potentially cheap source of biomass material for fermentation. The cardboard waste and mixed bio waste used for fermentations CA20-1 and BW20-2 were both enzymatically treated using amylases and cellulases by BioGasol ApS and subjected to Pentocrobe 411 fermentation as described above. Both materials contained significant amounts of lactic acid but relatively low levels of acetic acid and furfural and both contained significant amounts of xylose. The total sugar concentration was below 75 g/L for both materials and it was therefore not necessary to use nitrogen sparging.

The cardboard and bio waste fermentations both showed modest ethanol productivities indicating that the lactic acid or other compounds in the waste biomasses may have been inhibitory. The ethanol yield was comparable to the other tested biomasses and the sugar conversion was high (98–99%). The final ethanol concentration of 23–35 g/L is limited by the sugar concentration of the original biomasses as almost undiluted biomasses were used in the fermentations.

### Tolerance of Pentocrobe 411 to 2-furaldehyde (furfural) and acetic acid in minimal medium

**Fig 5:**
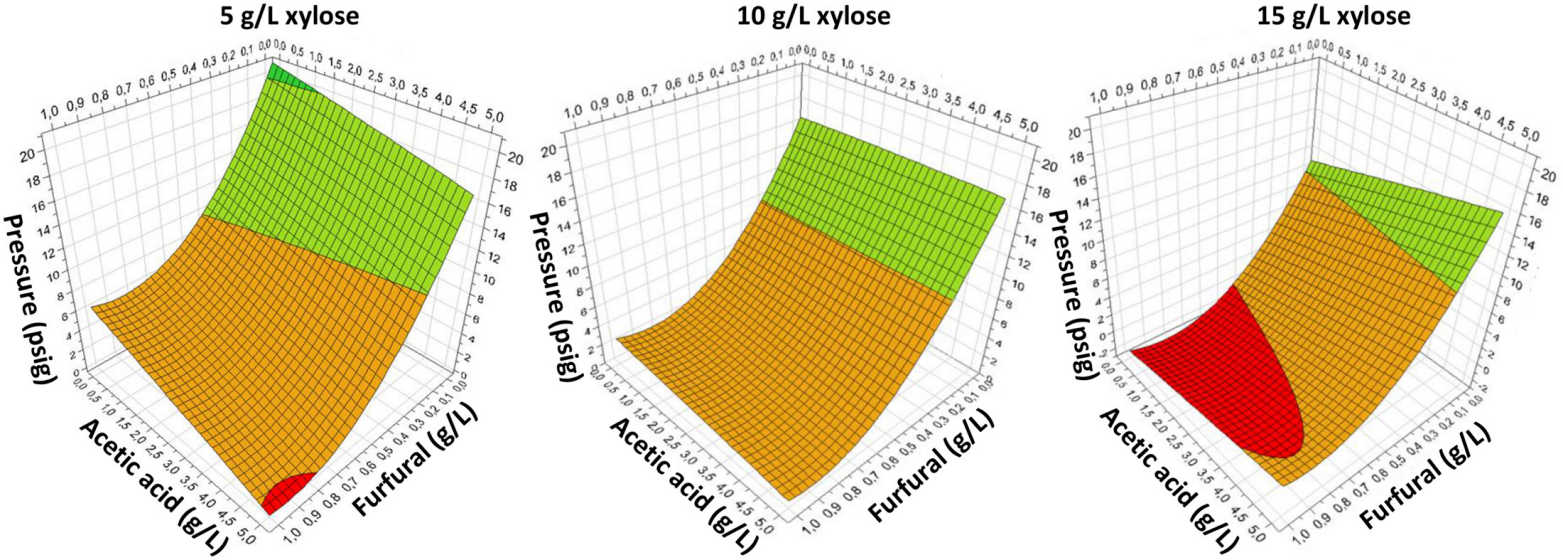
Statistical design of experiments showing the combined inhibitory effects of furfural (0–1 g/L), acetic acid (0–5 g/L) and xylose (5, 10, and 15 g/L). Pressure (psig) is an indicator of growth. Pressures above 10 psig (green zone) indicate that the fermentation is not inhibited.

*Thermoanaerobacter italicus* Pentocrobe 411X was tested in 10 mL anaerobic batch fermentations in minimal medium with varying concentrations of xylose, acetic acid and furfural (5–15 g/L, 0–6 g/L, and 0–1 g/L, respectively) in a statistical design experiment to test the effect of individual inhibitory components of the biomass on cell growth (Fig 5). Within the ranges tested, furfural had the greatest impact on growth. At concentrations above 0.5 g/L furfural, no growth was observed at any concentrations of xylose or acetic acid. A xylose concentration of 15 g/L apparently increased the inhibitory effect of furfural, whereas the concentration of acetic acid had no significant impact on the inhibitory effect of furfural when tested in the concentration range relevant to hydrolysates.

## Discussion

Lignocellulosic material is potentially the world’s largest source of fermentable sugars for biofuels and chemicals, but to become a significant contributor to the world fuel production, ethanol derived from lignocellulosic material must be produced efficiently and with high yields. Fermentation performance is one of the pieces of this puzzle that must be optimized, not only separately, but also in relation to pretreatment, enzymatic hydrolysis and downstream processing. Over the last 30 years, hundreds of different bacteria and yeasts have been proposed as candidates for fermentation of lignocellulosic biomasses [11–13].

The data presented here demonstrate that *Thermoanaerobacter* Pentocrobe 411 is capable of achieving high ethanol yields and productivities on a broad range of biomasses including wheat straw, birch, corn cob, sugar cane bagasse, oil palm empty fruit bunches and frond, mixed waste from food production and cardboard in high temperature continuous fermentation systems. In addition, cane syrup and cane molasses were efficiently fermented. Ethanol yields were between 0.47–0.49 g/g (92%-96% of theoretical maximum) when based on glucose, xylose and arabinose and between 0.47 and 0.51 g/g (92%-100% of theoretical) when based on glucose and xylose. As most genetically modified yeasts do not consume arabinose, the most common basis for comparison is glucose and xylose only. The volumetric ethanol productivity varied from 1.2 g/L/h for biowaste to 2.7 g/L/h for cane molasses and the final ethanol concentration achieved varied from 23 g/L (cardboard) to 104 g/L (cane molasses). The difference in the maximal productivity is likely to be caused by the difference in the concentration of inhibitory compounds relative to the sugar concentration. Inhibitors such as acetic acid cause the cells to generate more ATP in order to maintain intracellular pH thereby forcing the cell to produce ethanol at the expense of cell growth [14]. The reduced cell growth sets a lower limit to the hydraulic retention time allowed if wash-out is to be avoided and, as the reactor is controlled on the basis on sugar conversion, the result is therefore an upper limit to the ethanol productivity. The maximal growth rate of Pentocrobe 411 is approximately 0.3 h^-1^ in minimal medium with no inhibitors present [9] and a minimal hydraulic retention time is therefore expected to be in the range of 4–5 hours. The lowest hydraulic retention time achieved in the current fermentations was 12 hours in the relatively low sugar WS80 fermentation, while the highest ethanol productivity was achieved on sugar cane molasses, containing a low concentration of inhibitors and a high concentration of sugar.

None of the biomass hydrolysates used in this study were detoxified using e.g. overliming and significant amounts of inhibitors were therefore present. As can be seen from Table 3, the maximal levels of acetic acid, lactic acid and furfural applied to the fermentations were 6.2 g/L, and 12.3 g/L, and 0.88 g/L respectively (Table 3).

**Table 3:** Concentration of acetic acid, lactic acid and furfural in the final and most concentrated influents applied to the fermentations (g/L).

	WS80	WS97-N2	WS10	BI13B	CC02-N2	SB10B	CA20-1	BW20-2	EFB16-1-N2	FR16-2-N2	CS02B-N2	CM02B-N2
Acetic acid	4.89	6.18	2.65	4.19	4.39	0.39	0.44	1.95	5.02	3.62	0.20	0.74
Lactic acid	0.00	0.00	0.00	0.00	0.37	9.82	12.3	6.81	0.33	0.00	1.76	3.21
Furfural	0.41	0.35	0.88	0.14	0.29	0.69	0.09	0.00	0.67	0.72	0.00	0.00

The names of the biomass influents (WS80, WS97-N2 etc.) relate to the compositions shown in Table 1.

*Thermoanarobacter italicus* Pentocrobe 411 is tolerant to up to 38 g/L (0.46 M) sodium acetate and up to around 0.5 g/L of furfural when this is added to defined medium in batch culture (S2 Fig, Fig 5). However, furan derivatives such as furfural are known to act synergistically with other inhibitory compounds including phenolic compounds, and acetic, formic and levulinic acids [14] all of which are present in the lignocellulosic biomasses, and the medium may therefore be inhibitory even if acetic acid and furfural are not at a critical level (e.g. Table 3, WS80). Comparing Table 3 to Fig 5, the maximum limit to the concentration of pretreated biomass can be explained by the combined inhibitory effect of furfural and acetic acid for the media containing wheat straw, sugar cane bagasse, empty fruit bunch, and oil palm frond. For birch wood, corn cob, cardboard, biowaste, cane syrup, and cane molasses, other inhibitors such as lignin degradation products, lactic acid, waxes, and inorganic salts may be contributing to the inhibitory effect since acetic acid and furfural are both relatively low in the final fermentation influents.

Furfural has been shown to be a potent inhibitor of both bacteria and yeast fermentation and the high hydrophobicity of furfural has been shown to lead to cell membrane disruption, causing a reduction in cell replication rate, ATP production, and inhibition of glycolytic and fermentative enzymes in central metabolic pathways [14–15]. *Thermoanaerobacter* is at least five-fold more tolerant to biomass hydrolysates when grown in continuous fermentation as opposed to batch fermentation (S3 Fig compared to Table 3, WS80). This had previously been attributed to immobilization of the organisms in continuous culture [4], but as the current study has demonstrated high tolerance in non-immobilized reactor systems, this view will have to be revised. HPLC analysis shows non-detectable furfural levels in the reactor effluents indicating that furfural is converted during the continuous fermentation. *Thermoanaerobacter pseudoethanolicus* 39E has been shown to possess an NADH dependent alcohol dehydrogenase that reduces furfural to the less toxic furfuryl alcohol leading to reduced toxicity, a pathway that is also known to be present in yeast and other bacteria [16]. In batch culture, the microorganism will be presented with the full concentration of furfural from the beginning of the fermentation whilst in the continuous fermentation the biomass medium is gradually introduced allowing the cell to increase the expression of the relevant alcohol dehydrogenase and thereby to prevent the full effect of the furfural.

Increasing reactor ethanol concentration beyond 25–30 g/L inhibits cell growth and nitrogen sparging was therefore introduced when fermenting on medium with sugar concentrations above 70–75 g/L to increase the natural evaporation of ethanol in the high temperature fermentations. In industrial fermentations, nitrogen sparging may be economically prohibitive and alternative solutions may be used instead. Because of the high temperature of the fermentation, only a modest vacuum would be necessary to remove significant amounts of ethanol from the broth for instance in a recirculation loop connected to the reactor. Pentocrobe 411 has been shown to be tolerant to both vacuum and to increased pressure as long as anaerobic conditions are maintained.

The high temperature of the fermentations (66°C) is not only beneficial with respect to removal of surplus ethanol but is also important for the prevention of contamination. As shown in the WS97-N2 wheat straw fermentation, influents can be contaminated with for instance lactic acid bacteria leading to decreased yields and productivities. However, the data also show that as soon as the influent was changed, the lactic acid level decreased in the reactor even though no attempts were made to decontaminate the main reactor. Particularly for continuous fermentations it is an important advantage that decontamination of the main fermentor can be reduced to a minimum.

This study shows that *Thermoanaerobacter* Pentocrobe 411 can efficiently convert glucose, xylose and arabinose from a broad range of different biomasses with yields close to the theoretical maximum. The ability of strains of the *Thermoanaerobacter* genus to co-ferment hexoses and pentoses is well known from literature [17–19]. Being a strictly anaerobic microorganism, *Thermoanaerobacter* lacks an oxidative pentose phosphate pathway (PPP) for converting hexoses to pentoses. Consequently, any preference for hexose would have compromised the many crucial metabolic processes where pentoses play essential roles [18]. Here, the co-utilization of glucose, xylose, and arabinose is demonstrated in continuous culture using pretreated lignocellulosic substrates. In media where the glucose concentration is low such as with the x wheat straw pentose fraction (WS80), the conversion of xylose is even observed to exceed that of glucose. The ability to use very different biomasses under similar conditions makes it possible to base a bioethanol plant on a variety of local biomasses which will reduce the plant transport radius and will decrease the risk of local increase in feedstock prices due to limited biomass availability.

*Thermoanaerobacter* Pentocrobe 411 is deposited in an open strain collection (DSMZ, Germany) under accession number DSM 29083, to allow a wider research into the possibilities of thermophilic bacteria for ethanol production. As the complicated process steps of lignocellulosic ethanol production are highly intertwined and the current economy of the overall process is just bordering on profitability, testing and collaboration of stains across fields is a necessity for a successful outcome. In contrast to yeast, the major challenge to be overcome for thermophilic bacteria as an industrial fermentation organism is not related to changes to sugar metabolism or to inhibitor tolerance but rather to the demonstration of performance in pilot and demonstration scale of the more complicated continuous fermentation systems.

## Supporting Information

S1 Fig Fermentation system. The pictures show the laboratory setup used for the fermentations including the feed bottle (1), feed pump (2), fermentor (3), NaOH pump (4), NaOH flask (5), pH and temperature control (6), effluent bottle (10), and waterbath (11). The influent enters through a needle at the top of the fermentor and effluent exit from the overflow outlet and into the effluent bottle. Gas exits with the effluent. Mixing is achieved with a magnetic stirrer. The pH is maintained at 7.0 automatically by controlling the NaOH pump based on readings from the combined temperature and pH sensor in the reactor.
(TIFF)

S2 Fig Tolerance of *Thermoanaerobacter* Pentocrobe 411 to sodium acetate. Gas pressure (psig) in the headspace of growing cultures of *Thermoanaerobacter* Pentocrobe 411 at day 1 and day 2 after inoculation in 10 mL BA medium with varying concentrations of sodium acetate in 20 mL anaerobic closed tubes. Increasing headspace pressure is linked to production of ethanol, since 1 mole of CO2 is produced for each mole of ethanol. If no salts are added, a pressure of 10–15 psig is reached after 1 day of fermentation.
(TIFF)

S3 Fig Tolerance of *Thermoanaerobacter* Pentocrobe 411 to pretreated wheat straw liquid fraction. Gas pressure (psig) in the headspace of growing cultures of *Thermoanaerobacter* Pentocrobe 411 after inoculation in 10 mL BA medium with 5 g/L xylose and varying concentrations of the liquid fraction from pretreated wheat straw (adjusted to pH 7) in 20 mL anaerobic closed tubes. The concentration of furaldehyde (2-fur), acetate, and total sugars from the pretreated wheat straw is shown below the columns. The pressure of each tube was measured after 24, 48 and 72 hours. The decreasing pressure observed in the control tubes (first three bars) is due to loss of pressure during the measurement.
(TIFF)
