# Supplementary figures and images for: Continuous Ethanol Fermentation of Pretreated Lignocellulosic Biomasses, Waste Biomasses, Molasses and Syrup Using the Anaerobic, Thermophilic Bacterium Thermoanaerobacter italicus Pentocrobe 411
Source: PLoS One. 2015 Aug 21;10(8):e0136060. doi: 10.1371/journal.pone.0136060 (PMC4546601; doi:10.1371/journal.pone.0136060)

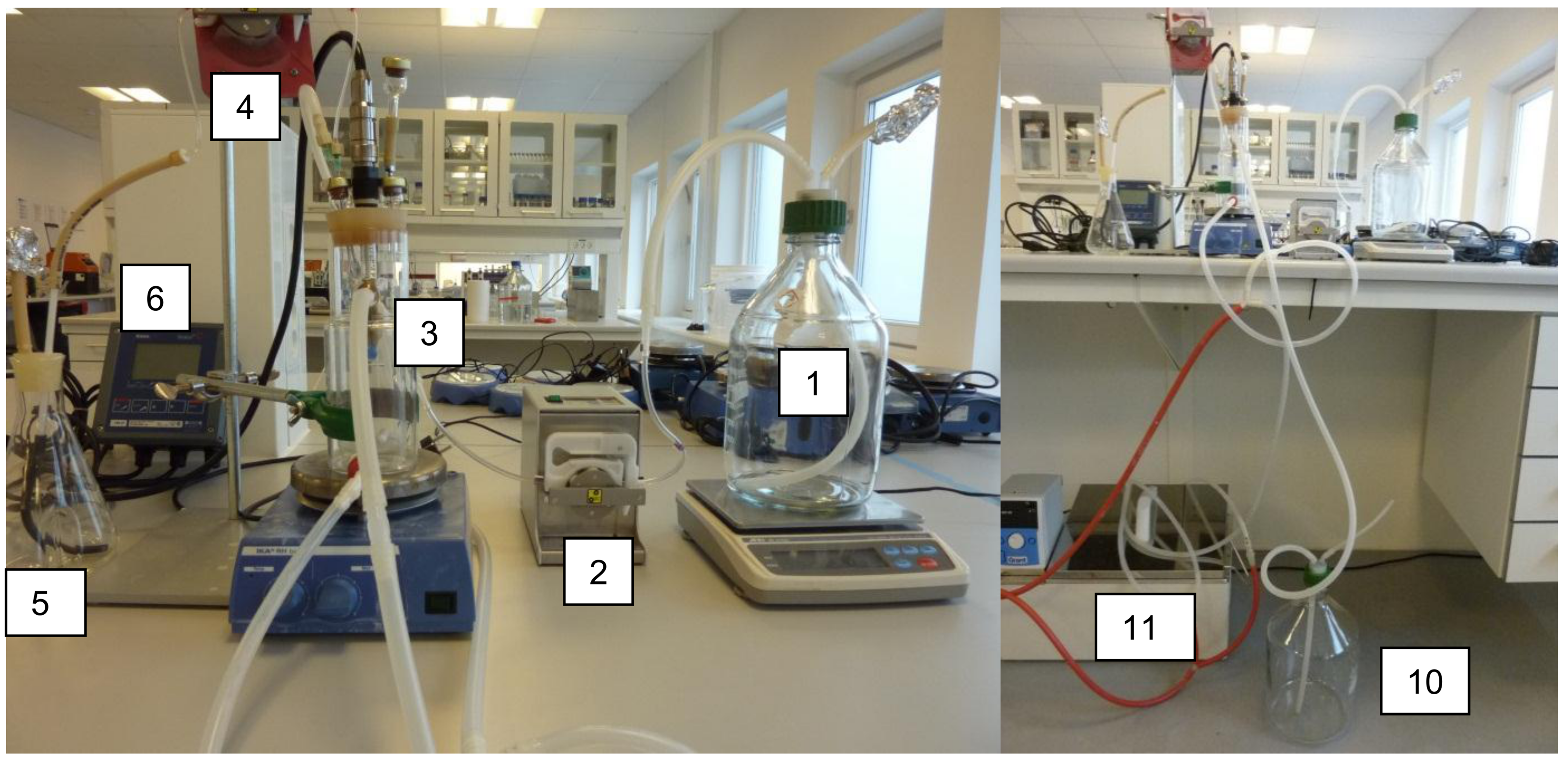

Supplement: S1 Fig — The pictures show the laboratory setup used for the fermentations including the feed bottle (1), feed pump (2), fermentor (3), NaOH pump (4), NaOH flask (5), pH and temperature control (6), effluent bottle (10), and waterbath (11). The influent enters through a needle at the top of the fermentor and effluent exit from the overflow outlet and into the effluent bottle. Gas exits with the effluent. Mixing is achieved with a magnetic stirrer. The pH is maintained at 7.0 automatically by controlling the NaOH pump based on readings from the combined temperature and pH sensor in the reactor. (TIFF) [file pone.0136060.s001.tiff]

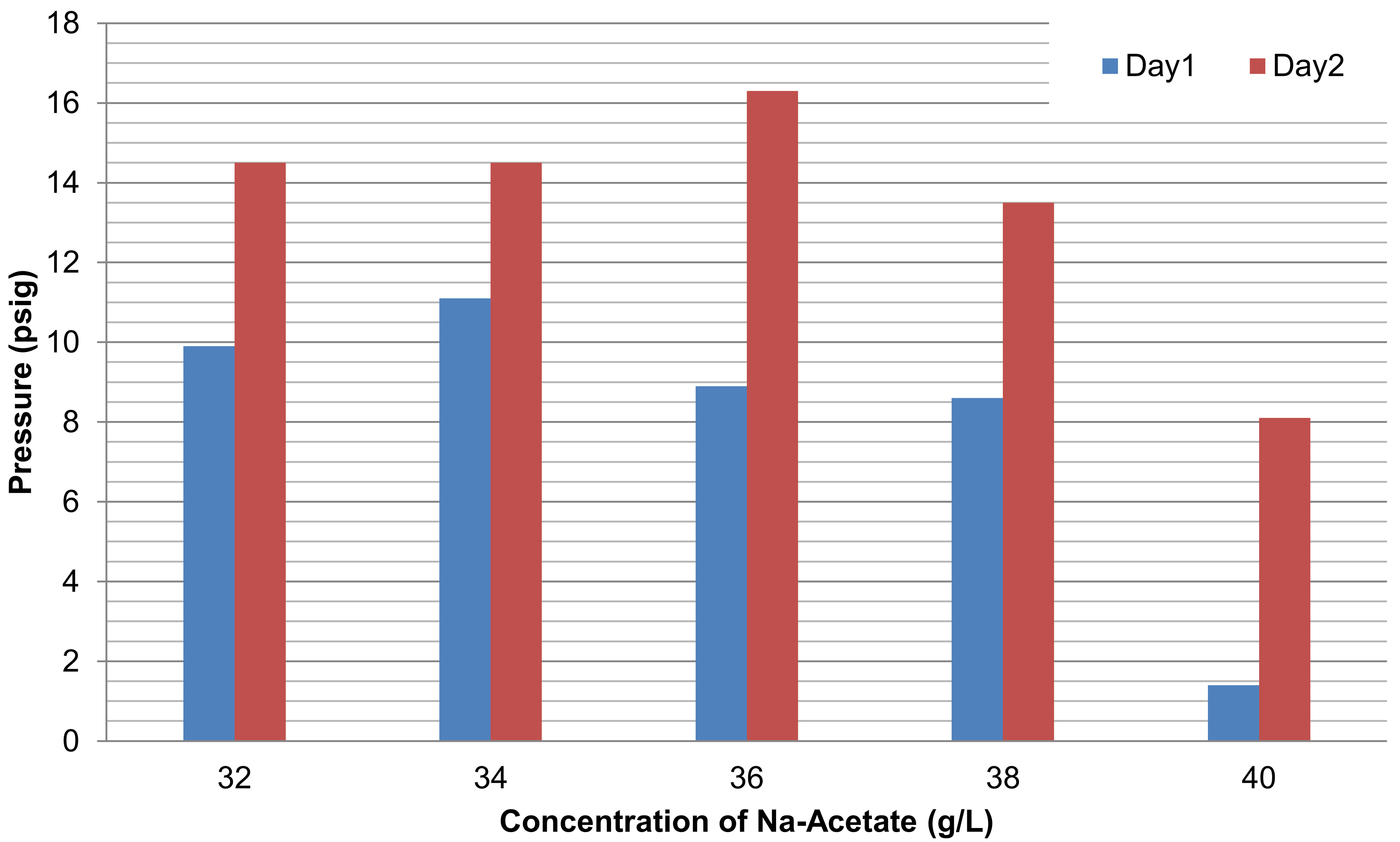

Supplement: S2 Fig — Gas pressure (psig) in the headspace of growing cultures of Thermoanaerobacter Pentocrobe 411 at day 1 and day 2 after inoculation in 10 mL BA medium with varying concentrations of sodium acetate in 20 mL anaerobic closed tubes. Increasing headspace pressure is linked to production of ethanol, since 1 mole of CO2 is produced for each mole of ethanol. If no salts are added, a pressure of 10–15 psig is reached after 1 day of fermentation. (TIFF) [file pone.0136060.s002.tiff]

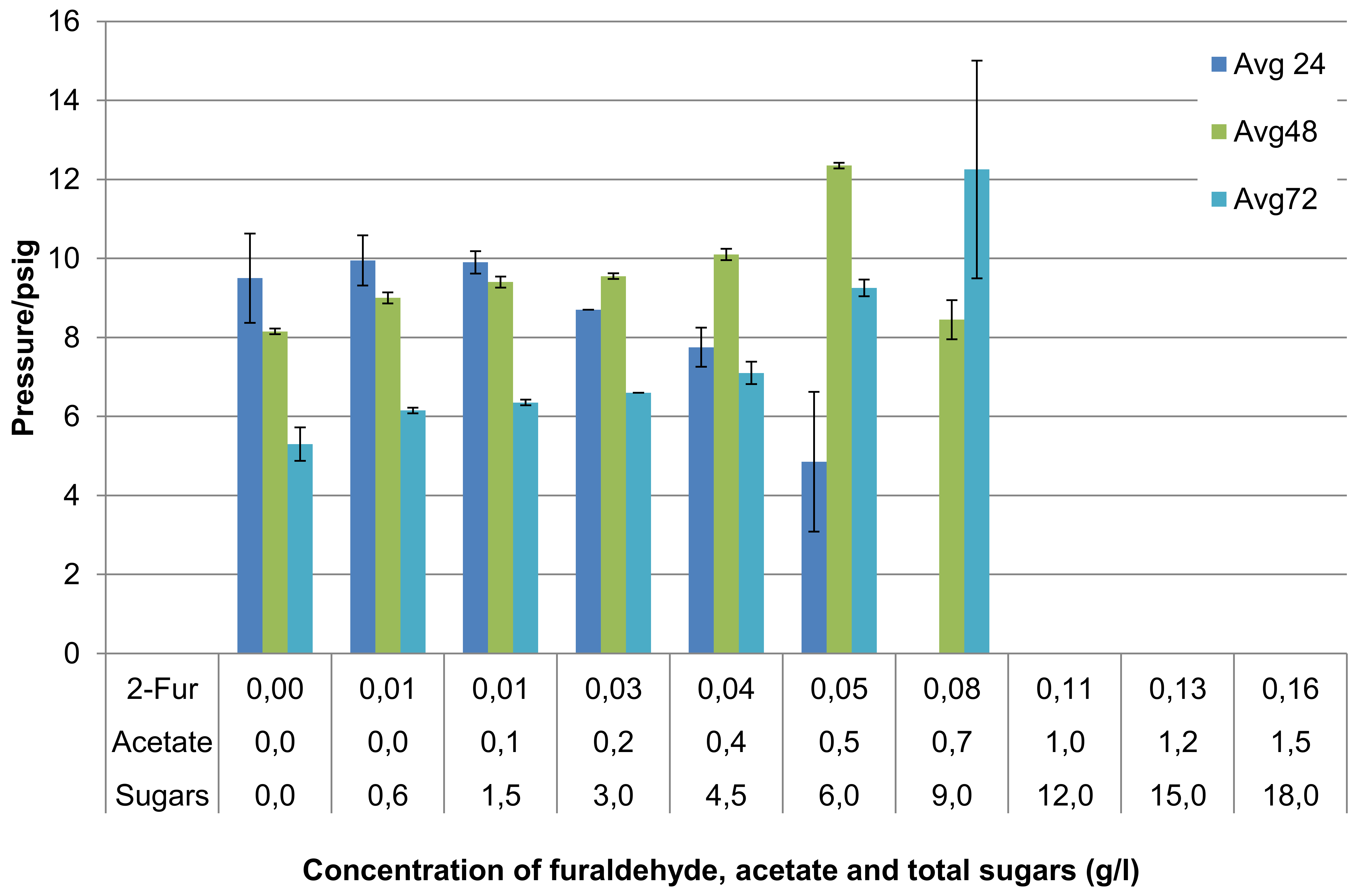

Supplement: S3 Fig — Gas pressure (psig) in the headspace of growing cultures of Thermoanaerobacter Pentocrobe 411 after inoculation in 10 mL BA medium with 5 g/L xylose and varying concentrations of the liquid fraction from pretreated wheat straw (adjusted to pH 7) in 20 mL anaerobic closed tubes. The concentration of furaldehyde (2-fur), acetate, and total sugars from the pretreated wheat straw is shown below the columns. The pressure of each tube was measured after 24, 48 and 72 hours. The decreasing pressure observed in the control tubes (first three bars) is due to loss of pressure during the measurement. (TIFF) [file pone.0136060.s003.tiff]
